# Hereditary gingival fibromatosis: Clinical and ultrastructural features of a new family

**DOI:** 10.4317/medoral.20170

**Published:** 2014-12-05

**Authors:** Sabina-Pena-Borges Pego, Ricardo D. Coletta, Danilo-Cangussu Mendes, Paulo-Rogério de Faria, Mário R. Melo-Filho, Lucas-Rodrigues Alves, Hercílio Martelli-Júnior

**Affiliations:** 1 Stomatology Clinic, Dental School, State University of Montes Claros, Montes Claros, Minas Gerais, Brazil; 2 Department of Oral Diagnosis, School of Dentistry, State University of Campinas, Piracicaba, São Paulo, Brazil; 3 Institute of Biomedical Science, Department of Morphology, University of Uberlandia, Uberlandia, Minas Gerais, Brazil

## Abstract

Objective: This article describes the diagnosis, clinical and microscopic (histopathology and ultrastructural) features and treatment of a new family with hereditary gingival fibromatosis (HGF) and highlights the importance of this genetic condition. Study Design: To characterize the pattern of inheritance and the clinical features, members of a new family with HGF were examined. The pedigree was reliably constructed including the four latest generations of family. Hematoxylin and eosin staining and ultrastructural analysis were performed with the gingival tissue. Results: Examination of the family pedigree revealed that the patient III-2 represent the index patient of this family (initial patient with a mutation), which was transmitted to her daughter through an autosomal dominant mode of inheritance. The affected patients showed a generalized gingival overgrowth. The patient was treated with surgical procedures of gingivectomy and gingivoplasty. The diagnosis was confirmed by histopathology examination that showed a well-structured epithelium with elongated and thin papillae inserted in fibrous connective tissue with increased amount of collagen. The ultrastructural aspects of the tissue show collagen fibrils exhibiting their typically repeating banding pattern with some fibrils displaying loops at their end. Moreover, it was possible to seen in some regions fibrillar component presenting tortuous aspects and loss of the alignment among them. Conclusions: This HGF frequently resulted in both esthetic and functional problems. The genetic pattern of this Brazilian family suggested a new mutation, which was later transmitted by an autosomal dominant trait.

** Key words:**Gingival fibromatosis, genetic disease, pedigree, ultrastructure.

## Introduction

Gingival fibromatosis (GF) is the overgrowth of the gingival characterized by the expansion and accumulation of the connective tissue with the occasional presence of an increased number of cells (1). It is a hereditary condition or due to side effect of medications including phenytoin, cyclosporin, and nifedipine (2). The inherited form called hereditary gingival fibromatosis (HGF, OMIM 135300) is a rare isolated condition manifested by slowly progressive, benign, localized or generalized enlargement of the gingival with an incidence of 1:750,000 live births (2-4).

The gingival enlargement is of normal color, firm consistency, non-hemorrhagic and asymptomatic. Genders are equally affected (5). Its overgrowth of gingiva results in both esthetic and functional problems for affected individuals. The most common effects are diastemas, malpositioning of teeth, prolonged retention of primary dentition, delayed eruption, cross and open bites, prominent lips, and open lip posture (2,6). Although the gingival enlargement does not directly affect the alveolar bone, the gingival swelling may increase the bacterial plaque accumulation, leading to periodontitis, bone resorption and halitosis (2).

Most cases of HGF are detected at birth, but sometimes it may not be noted until later childhood, at the time of eruption of deciduous or permanent teeth (7). The histopathology features of HGF reveals dense connective tissue rich in collagen fibers and a hyperplasic epithelium with long rete pegs (8). Small calcified particles, islands osseous metaplasia, ulceration of the overlying mucosa and inflammation can also be observed occasionally (9).

This article describes the diagnosis, clinical and microscopic (histopathology and ultra structural) features and the treatment of a new family with HGF and highlights the importance of this genetic condition.

## Material and Methods

The study protocol was approved by the Institutional Research Ethics Committee. Informed consent was taken from the parents or the legal guardians of the children.

The pedigree was reliably constructed including the four latest generations of family. Hematoxylin and eosin staining and ultrastructural analysis were performed from gingival tissue.

A small fragment of gingiva was also obtained to be analyzed on electron microscopy (Zeiss EM 109 – MegaviewG2/Olympus Soft Imaging Solutions). Briefly, the gingival sample was fixed using the standard fixation protocol of the glutaraldehyde-osmium tetroxide, which was followed by dehydration in acetone and infiltration with epoxy resin. Ultrathin sections of 60 nm in thickness were made and then stained first with uranyl acetate and then lead nitrate.

## Results

- Pedigree Analysis

**Figure 1:**
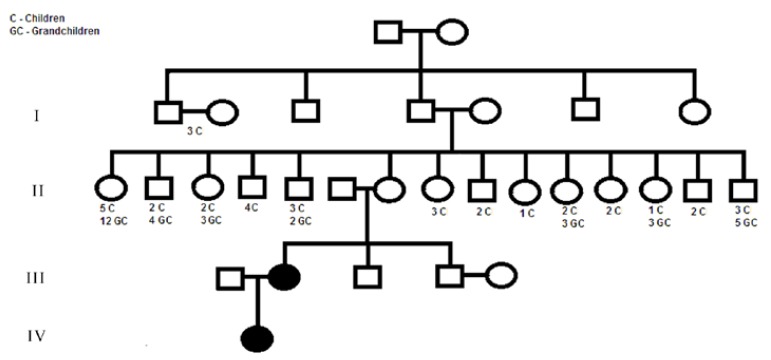
Pedigree of family affected with Hereditary gingival fibromatosis, showing an autosomal dominant trait. Affected individuals are indicated by blackened symbols; squares denote males and circles denote females.

The pedigree was reliably constructed including the four latest generations and is depicted in figure 1. The clinical examination and history of this family revealed that only two members (2.22%) presented a generalized gingival overgrowth. Genetic abnormalities commonly associated with GF, characterizing a syndrome, were not identified in this family. In the third generation, there was one affected member, who transmitted the trait to his descendant (family proband) (fourth generation). No history of consanguinity was verified in the family. Thus, patient III-2 represent the index patient of this family (initial patient with the mutation), which was transmitted to her daughter probably through an autosomal dominant mode of inheritance. Following is a brief clinical description of the two patients with HGF.

- Clinical Analysis

A three-year-old Caucasian girl was referred to the Stomatology Clinic of the State University of Montes Claros, due to pronounced fibrous gingival overgrowth. The girl came accompanied by her maternal grandmother. Her medical history was unremarkable. Her weight and height were within normal range and she did not exhibit any mental impairment.

**Figure 2:**
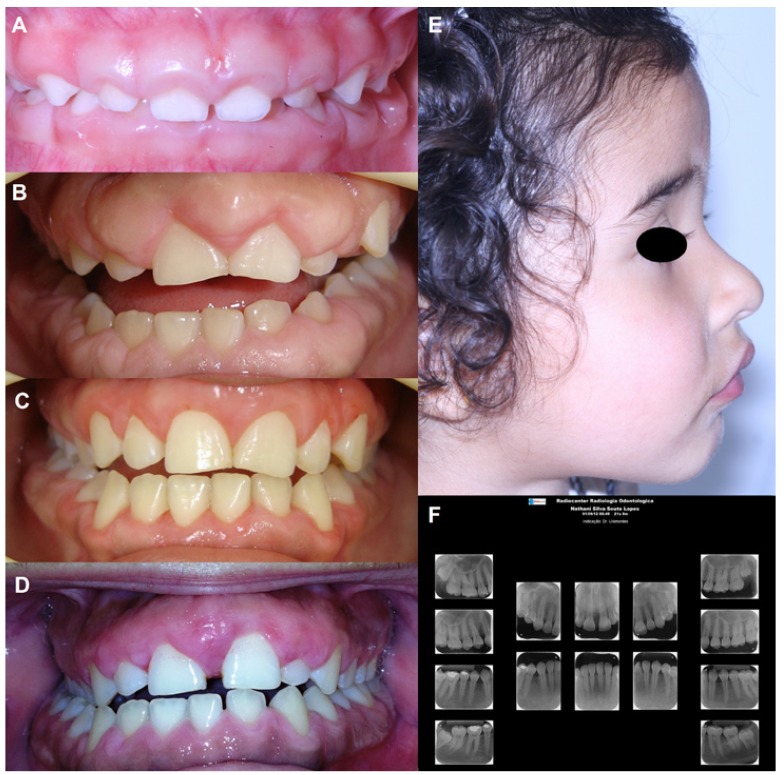
Clinical and radiographic characteristics of the family with Hereditary gingival fibromatosis. Gingival overgrowth in the anterior facial area of the proband (A) and her mother (B). In both, severe and generalized gingival enlargement involving both the maxillary and mandibular arches, covers almost two-thirds of the clinical crowns. The teeth are morphologically normal. (C) Aspect of gingiva after the last surgical intervention in the mother. (D). After seven years, the mother presents gingival overgrowth, indicating the recurrence of Hereditary gingival fibromatosis. (E) Proand had a marked cross bite with difficulties in the correct lip closure. (F) The panoramic radiography revealed any missing or malformed teeth of mother’s proband.

Clinical examination revealed generalized gingival hyperplasia involving both the maxillary and mandibular arches, with morphologically normal teeth. The gingiva was pink, with fibrous consistency and covering some dental crowns (Fig. 2). Gingival enlargement had been initially noticed two months after birth, but it became more intense during the period of deciduous dentition. She had a marked cross bite with difficulties in the correct lip closure (Fig. 2).

The grandmother reported that her daughter (mother of the proband) also exhibited gingival overgrowth. In the following visit, the girl’s mother came with her daughter. The mother has 21 years and her medical history was also unremarkable. The patient did not have any evidence of genetic syndrome, mental disorder or use of medications associated with gingival hyperplasia. Due to gingival overgrowth, she was submitted to several gingival surgeries seven years ago at the age of 14 years old (Fig. 2). Currently, the patient presents gingival overgrowth, indicating the recurrence of HGF (Fig. 2). Tooth malformations were not found. The radiographic examination did not show any missing or malformed teeth (Fig. 2).

- Treatment

After oral hygiene instruction, the surgical treatment consisted of quadrant- by-quadrant gingivectomy/gingivoplasty technique, followed by 0.12% chlorhexidine oral rinses twice a day for 2 weeks after each surgery. After the last surgical intervention, both patients periodically returned for follow-up visits. Therapy at the post surgical follow-up visit was a cosmetic and functional improvement. Scaling and prophylaxis continued to be performed every 6 months. The proband is being accompanied, with professional prophylaxis and later will be performed the surgeries of gingivectomy/gingivoplasty.

- Histopathology Analysis

**Figure 3:**
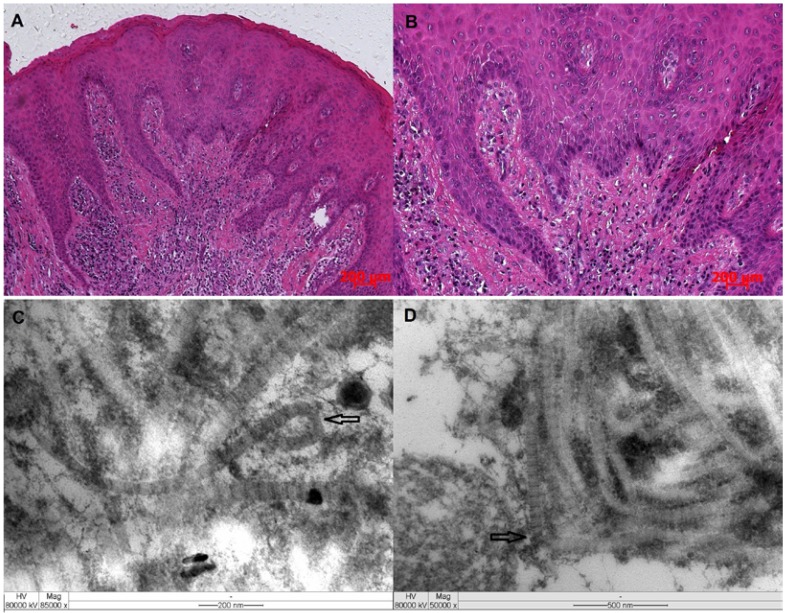
(A/B) Histologic morphology from gingival tissue of the mother’s proband showing an increased amount of collagen fiber bundles running in all directions and significant extension of the epithelial rete ridges (H&E; original magnification – x10 and x50). Ultrastructural aspects of the tissue revealed collagen fibrils showing loops (C, arrow) and other ones with apparent tortuous aspects and altered alignment among them (D, arrow).

The histologic examination of gingival overgrowth tissue specimens revealed a well-structured epithelium with elongated and thin papillae inserted in fibrous connective tissue. The connective tissue showed an increased amount of collagen fiber bundles running in all directions. A chronic inflammatory cell infiltrate was also observed (Fig. 3).

- Ultra structural Aspects

The ultra structural aspects of the tissue show collagen fibrils exhibiting their typically repeating banding pattern of with some fibrils displaying loops at their end. Moreover, an alignment among them was also observed in few regions (Fig. 3).

## Discussion

HGF is traditionally considered an autosomal dominant disease (10). Few reports have described that HGF may be inherited by an autosomal recessive gene (4,6,11). However, some of the reports in which HGF was transmitted as an autosomal recessive inheritance clearly demonstrated that the gingival overgrowth is associated with other systemic alterations as part of a syndrome (4,6,12). In cases where HGF is transmitted as a recessive trait without other associated features, the history of consanguinity in the family seems to be always present (13).

On the other hand, GF may be caused by a new mutation. In these cases, the individuals are diagnosed as idiopathic gingival fibromatosis since there was no evidence of genetic transmission in their family histories (2). The data described here provides evidence that the patient III-2 represents the index patient of this family, and she transmitted the genetic defect to her daughter via autosomal dominant mode of inheritance. However, it has been reported that unaffected individuals can occasionally transmit HGF in an autosomal dominant pattern to their offspring without themselves being clinically affected. As, HGF is very uncommon and there was no history of consanguinity in the family, an autosomal recessive mode of transmission is unlikely. X-linked inheritance is a possibility, but no reports of this type of Mendelian trait have been described in HGF (14). Phenotype characteristics suggestive of a genetic syndrome were also not observed in any member of this family.

The clinical manifestations of the cases reported here were consistent with many reports of HGF (4,5,11). Manifestations of HGF may include focal sites of gingival hyperplasia or generalized involvement, and the degree of hyperplasia may vary from slight to severe (4,5). The cases reported here exhibited generalized and gingival enlargement that inhibited the complete eruption of the deciduous or permanent dentition into the oral cavity. The anterior region of both maxilla and mandible were most severely affected. Histopathological aspects observed in this study showed gingival tissues with a well-structured epithelium with elongated and thin papillae inserted into deep fibrous connective tissue with collagen fiber bundles running in all directions. These microscopic findings are similar to those observed previously in other families (15,16). Ultrastructural aspects revealed collagen fibrils presenting loops at their edges and loss of packing along with an apparent tortuous pattern of organization and dissociation of them. On the other hand, an apparent changing in collagen fibrils’ diameter was not observed in the tissue sample analyzed here, although Barros *et al*. 2001 (17) had described this feature as an important ultrastructural finding to be observed in samples taken from hereditary gingival fibromatosis patient.

It is known from previous reports that the HGF usually begins at the time of eruption of the permanent dentition (4), but it can be detected at the eruption of the deciduous dentition and rarely at birth (11). The enlargement seems to progress rapidly during “active” eruption and decrease at the end of this stage (18). The presence of teeth appears to be necessary for HGF to occur, because the condition disappears or recedes with the loss of the teeth (19). In the proband of the present study, the alteration was firstly noticed two months after birth, but it became more intense during the period of deciduous dentition. In the proband’s mother, gingival enlargement developed with the eruption of permanent dentition. Indeed, HGF is clearly a heterogeneous disorder in regard to clinical presentation and genetic inheritance (3,20).

HGF cannot be cured but may be controlled with varying degrees of success. When the enlargement is minimal, thorough scaling of teeth and home care may be all that are required to maintain good appearance. However, excessive gingival tissue points to surgical intervention. Several authors have reported the recurrence of hyperplasic tissue in HGF following surgical treatment, but the psychological benefits of even temporary cosmetic improvement must not be underestimated and may outweigh the probability of recurrences in such a severe case (19). Here, the mother was unhappy with the appearance of their gingival and was surgically treated by a combination of gingivectomy and gingivoplasty. In this case, the recurrence was observed 7 years after initial treatment. In the proband, the approach was based on professional supervision and later the performance of surgical procedure to control gingival increase.

## Conclusion

In summary, we evaluated four generations of a Brazilian family with two individuals exhibiting HGF. Clinical assessment revealed generalized gingival hyperplasia involving both the maxillary and mandibular arches, with morphologically normal teeth and histopathological analysis of gingival overgrowth tissue specimens showed a well-structured epithelium with elongated and thin papillae inserted in fibrous connective tissue. The genetic pattern of this family suggested a new mutation, which was later transmitted by an autosomal dominant trait. Further genetic studies are needed to better understand this HGF pathogenesis and inheritance patterns.
